# Progeroid features in a patient with Malouf syndrome due to a rare *LMNA* variant: a case report and review of the literature

**DOI:** 10.20945/2359-4292-2024-0491

**Published:** 2025-07-22

**Authors:** Aslihan Pekmezci, Aydeniz Aydin Gumus, Ozge Polat Korkmaz

**Affiliations:** 1 Department of Endocrinology and Metabolism, Basaksehir Cam and Sakura City Hospital, University of Health Sciences, Istanbul, Turkey; 2 Department of Medical Genetics, Basaksehir Cam and Sakura City Hospital, University of Health Sciences, Istanbul, Turkey

**Keywords:** Laminopathies, Lipodystrophy, Progeria

## Abstract

Laminopathiesrepresent a rare group of genetic disorders affecting various organs and tissues, including the skin, muscles, adipose tissue, bone, and cardiovascular system. The *LMNA* gene, the most common pathogenic gene responsible for laminopathies, harbors variants that can lead to diverse clinical phenotypes, such as progeroid syndromes, lipodystrophies, muscular dystrophies, and cardiomyopathies. This report presents a case of a young female patient who presented with prediabetes, secondary amenorrhea, and secondary osteoporosis. A 28-year-old female presented to our clinic with complaints of amenorrhea and decreased bone mineral density. She exhibited pronounced facial abnormalities and underdeveloped secondary sexual characteristics. Laboratory investigations revealed hypergonadotropic hypogonadism, prediabetes and hyperlipidemia. Significant mitral annular calcification was revealed via echocardiography. Genetic analysis revealed a *de novo* variant in exon 1 of the *LMNA* gene. This case reveals a novel laminopathy overlapping with the clinical features of Malouf syndrome while also exhibiting additional progeroid features, representing a distinct laminopathy. Furthermore, unlike previously reported cases with this genotype, it does not correspond to a progeroid syndrome typically associated with LMNA variants. Additionally, this case report is accompanied by a review of the relevant literature.

## INTRODUCTION

Lamins A and C are intermediate filaments encoded by the *LMNA* gene and are localized within the inner nuclear membrane (^1^). These structural proteins, in conjunction with the cytoskeleton, maintain cellular shape and size while also interacting with chromatin and transcription factors, playing both structural and regulatory roles (^1^). Variants in the *LMNA* gene have been linked to more than ten complex disorders collectively termed laminopathies (^2^). However, the variability in clinical manifestations among individuals with similar variants and the coexistence of multiple laminopathy features in families underscore the genetic and phenotypic diversity of laminopathies, making genotype-phenotype correlations challenging (^3^).

Malouf syndrome, characterized by a combination of dilated cardiomyopathy and hypergonadotropic hypogonadism, exhibits significant phenotypic heterogeneity, as evidenced by clinical variability among reported cases (^4^-^6^). Herein, we describe a 28-year-old female who presented with hypergonadotropic hypogonadism, cardiac valvular calcification and valvulopathy, prediabetes, hyperlipidemia, and distinctive progeroid facial, skin, and skeletal features. Genetic testing revealed a heterozygous *LMNA* c.331G>A p.(Glu111Lys) variant in exon 1. This report describes the patient’s clinical findings in the context of previously published cases.

## CASE REPORT

A 28-year-old female presented to our clinic with complaints of amenorrhea and decreased bone mineral density (BMD). The patient had experienced spontaneous menarche at the age of 12; however, she had been amenorrheic for the past 10 years. She had been under follow-up care at an external center for low BMD and had received denosumab therapy for the last 7 years. Her height and weight were 141 cm and 33 kg, respectively, and there was no evidence of cognitive impairment. The patient was born to nonconsanguineous parents and had two healthy sisters.

On physical examination, she exhibited pronounced facial abnormalities, including micrognathia, a broad nasal base, and bilateral blepharoptosis, resulting in a progeroid facial appearance. A spinal deformity led to postural abnormalities. Subcutaneous fat depletion was observed in the upper and lower extremities. Neuromuscular examination revealed preserved muscle strength (5/5) in all extremities, with no focal muscle atrophy or fasciculations. The skin appeared dry, thin, and sclerodermatous. There were no signs of *acanthosis nigricans*, pigmentation changes, or telangiectasia. Her secondary sexual characteristics were underdeveloped, with breast development corresponding to Tanner stage 2 and sparse pubic and axillary hair.

Laboratory investigations revealed mildly elevated alanine aminotransferase (ALT: 39 U/L; reference: zero to 35 U/L), aspartate aminotransferase (AST: 40 U/L; reference: 10 to 35 U/L), and gamma-glutamyl transferase (GGT: 47 U/L; reference: 5 to 36 U/L) levels. Viral serologies were unremarkable, and antimitochondrial antibodies (AMAs) were negative. Abdominal magnetic resonance imaging (MRI) excluded hepatosplenomegaly and hepatosteatosis. Pelvic MRI revealed a reduced ovarian volume with a normal-sized uterus. Metabolic assessment revealed a fasting plasma glucose level of 108 mg/dL, a glycated hemoglobin (HbA1c) level of 6.0% (reference: 4.8 to 5.9%), a serum insulin level of 17.7 µIU/mL (reference: 2.6 to 24.9 µIU/mL), and a C-peptide level of 3.01 ng/mL (reference: 1.1 to 4.4 ng/mL). Autoantibodies associated with diabetes, including anti-insulin, anti-glutamic acid decarboxylase (anti-GAD), and islet cell cytoplasmic antibodies, were negative. Genetic screening for maturity-onset diabetes of the young (MODY) was unremarkable. Lipid profiling revealed hypertriglyceridemia (214 mg/dL; reference: < 150 mg/dL). Autoimmune markers, including antinuclear antibodies (ANAs), anti-double stranded DNA (anti-dsDNA) antibodies, anti-centromere antibodies, and anti-topoisomerase 1 antibodies, were negative.

Given the patient’s appearance of subcutaneous fat loss in the upper and lower extremities, along with prediabetes and hyperlipidemia, a full-body composition analysis was performed using dual-energy X-ray absorptiometry (DXA) to test for lipodystrophy. The assessment revealed a total body fat percentage of 39.9%, a trunk-to-lower extremity fat ratio of 1.13, a trunk-to-limb fat mass ratio of 0.98, and a lower limb fat percentage of 47.46%. Additionally, the lower limb fat mass accounted for 30.57% of the total fat mass, indicating that there were no significant redistribution abnormalities. Consistently, pelvic MRI measurements revealed gluteal fat thicknesses of 19 mm on the right and 24 mm on the left, excluding the possibility of lipodystrophy. The serum leptin concentration was 5.45 ng/mL (reference: 0.7 to 9.1 ng/mL). Collectively, these findings suggest that the patient did not exhibit a lipodystrophic pattern.

Regarding neuromuscular assessments, DXA revealed a markedly reduced appendicular lean mass index (ALMI) of 3.6 kg/m^2^, indicating significantly decreased muscle mass. Despite this, muscle strength was fully preserved, with no signs of focal atrophy or fasciculations. The results of functional assessments, including gait speed and the chair stand test, were within normal limits, and sensory, coordination, and reflex examinations revealed no abnormalities. The creatine kinase (CK) level was 74 U/L (reference: zero to 170 U/L). Electrophysiological studies, including electromyography (EMG) and nerve conduction studies (NCSs), revealed normal motor and sensory conduction parameters, with no evidence of polyneuropathy, axonal degeneration, or demyelination.

The patient was undergoing denosumab therapy initiated at an external center, and her most recent DXA evaluation revealed a total lumbar Z score of -1.6 and a femoral neck Z score of -2.2, indicating a low bone mass for her age. A scoliosis curve with right convexity was evident on the radiographs. No findings of acroosteolysis or clavicular hypoplasia were observed. The vitamin D level was suboptimal (25-hydroxy vitamin D: 22 ng/mL), and other bone metabolism markers, including parathyroid hormone (PTH: 38 pg/mL; reference: 15 to 65 pg/mL), calcium (8.2 mg/dL; reference: 8.4 to 10.2 mg/dL), and phosphorus (4.5 mg/dL; reference: 2.5 to 4.5 mg/dL), were within the normal range. Owing to the patient’s secondary amenorrhea, hormonal evaluation was conducted, revealing elevated follicle-stimulating hormone (FSH) at 118 IU/L and luteinizing hormone (LH) at 61.9 IU/L levels and an undetectable estradiol level (< 5 ng/dL). Evaluation of other anterior pituitary hormones revealed no abnormalities.

Over the past 2 months, the patient reported progressively worsening exertional dyspnea and fatigue. There was no family history of sudden cardiac death. On physical examination, her blood pressure was 108/71 mmHg, and her heart rate was 130 beats per minute. Electrocardiography (ECG) demonstrated sinus tachycardia. Transthoracic echocardiography (TTE) revealed an ejection fraction of 60%, moderate aortic regurgitation, a calcified aortic valve with mild-to-moderate stenosis, and severe mitral stenosis with annular calcification extending into the mitral valve annulus. On the basis of these findings, transesophageal echocardiography (TEE) was performed, which confirmed severe mitral stenosis (valve area: 1 cm^2^), significant mitral annular calcification, moderate mitral regurgitation, grade 2 aortic regurgitation, and a calcified aortic valve. The patient was referred to the Cardiology Department for further management, where beta-blocker therapy and as-needed diuretics were initiated. The Cardiovascular Surgery Department recommended surgical intervention for valve replacement.

The patient, who presented with hypergonadotropic hypogonadism, low BMD, cardiac involvement, prediabetes, hyperlipidemia, and facial dysmorphism, was referred for genetic evaluation. Karyotyping revealed a 46XX chromosomal pattern. For clinical exome sequencing, next-generation sequencing (NGS) was performed using the MGI DNBSEQ-G400 platform, which employs DNA nanoball-based sequencing technology to generate high-throughput data. The raw sequencing reads were obtained in FASTQ format for subsequent bioinformatic analysis. Clinical exome sequencing of DNA isolated from a peripheral blood sample identified a heterozygous c.331G>A p.(Glu111Lys) variant in the *LMNA* gene. On the basis of these findings, the patient was diagnosed with Malouf syndrome. Genetic testing, including clinical exon sequencing, was also conducted on DNA samples from her parents and two sisters; however, no *LMNA* variants were identified in any of the family members, confirming that the variant occurred *de novo* in the patient.

The patient was started on dietary modifications alongside 15 mg pioglitazone to manage her metabolic dysfunction. For low BMD, she was prescribed calcium carbonate + cholecalciferol (1,000 mg/880 IU) effervescent tablets and vitamin D supplementation. Hormone replacement therapy with a combination of 2 mg estradiol valerate and 0.5 mg norgestrel was initiated to manage her hypergonadotropic hypogonadism and secondary osteopenia. Following combination therapy, the patient achieved a regular menstrual cycle. Subsequent treatment adjustments resulted in normalization of the HbA1c level, liver function tests, and triglyceride level. However, owing to her existing cardiac condition, pioglitazone therapy was discontinued and she was managed with dietary modifications alone for metabolic control. Metabolic stability was maintained solely through dietary adjustments, with no further deterioration observed in her metabolic parameters.

## DISCUSSION

Malouf syndrome, defined as the coexistence of hypergonadotropic hypogonadism and dilated cardiomyopathy, has been reported in various patients since 1973 (**Table 1**). The association between dilated cardiomyopathy and hypogonadism with *LMNA* variants was first described in 2003 by Chen and cols., who studied 26 patients with atypical Werner syndrome exhibiting progeroid features but lacking RECQL2 variants. They identified a heterozygous variant (A57P) in the *LMNA* gene in a 23-year-old Iranian female with lipodystrophy (^7^). In 2009, McPherson and cols. reported two additional cases. The shared clinical features of these three patients included hypogonadism, dilated cardiomyopathy, partial lipodystrophy, micrognathia, facial and skeletal abnormalities, and a progeroid appearance without alopecia or progressive atherosclerosis. These features have been individually reported in other laminopathies associated with *LMNA* variants. However, the combination of these overlapping features did not align with any single established laminopathy. In 2009, McPherson and cols. proposed that variants in exon 1 of the *LMNA* gene, which encodes the “rod domain” of the lamin protein, could lead to laminopathy clinically resembling Malouf syndrome due to the combined presence of dilated cardiomyopathy and hypergonadotropic hypogonadism (^8^).

**Table 1:** Clinical features of cases reported as Malouf syndrome in the literature

	Affected family members	Age at examination	Clinical features	Additional features
Narahara and cols. (^4^)	A sporadic case in her family	18 years	Dilated cardiomyopathy and hypergonadotropic hypogonadism	Mild mental retardation, broad nasal base, blepharoptosis, and minor skeletal abnormalities (arachnodactyly and mild thoracic scoliosis)
Malouf and cols. (^5^)	2 sisters	20 and 26 years old	Cardiomyopathy and hypergonadotropic hypogonadism	Bilateral ptosis and prominent nasal bone
Gursoy and cols. (^6^)	3 siblings (1 brother and 2 sisters	19 years old (male) and two older sisters	Cardiomyopathy and hypergonadotropic hypogonadism	Thyroid hemiagenesis in male sibling
Chen and cols. (^7^)	A sporadic case in her family	23 years old	Dilated cardiomyopathy and hypogonadism	Sloping shoulders, progeroid features (short stature, scleroderma-like skin, graying-thinning of hair) and lipodystrophy and osteoporosis
McPherson and cols. (^8^)	A sporadic case in her family	10 years old	Cardiomyopathy and hypergonadotropic hypogonadism	Progeroid features, lipodystrophy, osteopenia, sloping shoulders and clavicular hypoplasia
Najjar and cols. (^9^)	3 male siblings	2,5 years old, 5 days and 4 years old	Cardiomyopathy and hypoplastic genitalia	Mental retardation
Sacks and cols. (^10^)	3 male siblings	48, 46 and 29 years old	Cardiomyopathy and hypergonadotropic hypogonadism	Collagenoma
Najjar and cols. (^11^)	2 brothers	2,5 years old and at birth	Cardiomyopathy and hypoplastic genitalia	
Warren and cols. (^12^)	2 brothers	33 and 19 years old	Dilated cardiomyopathy and primary hypogonadism	Diabetes mellitus, hypertriglyceridemia, blindness and deafness
Thomas and cols. (^13^)	2 brothers	5 weeks and stillborn	Cardiomyopathy and hypergonadotropic hypogonadism	
Nguyen and cols. (^14^)	A sporadic case in her family	17 years old	Dilated cardiomyopathy McPhersona and cols. (^8^) restudied the patient and reported hypergonadotropic hypogonadism	Joint contractures, osteoporosis, skin findings (telangiectases, sclerodactyly, poikiloderma, soft tissue calcification) and facial features (small ears, narrow beaked nose, small chin)
Silfeler and cols. (^15^)	2 separate cases	25 and 22 years old	Cardiomyopathy and hypergonadotropic hypogonadism	
Present case	A sporadic case in her family	28 years old	Hypergonadotropic hypogonadism	Osteoporosis, cardiac valvular involvement and progeroid features (short stature, facial and skin features), scoliosis

The variant identified in our case also resides in exon 1 of the *LMNA* gene and shares phenotypic similarities with these three cases, as well as overlapping features with previously reported Malouf syndrome cases. However, our patient presented distinct differences, notably, the absence of lipodystrophy and the presence of advanced valvular calcification, which further differentiated this case from those previously described.

The most striking difference in the case we present, compared with previously reported cases of Malouf syndrome, is the prominence of progeroid features. These included short stature, scoliosis, metabolic dysfunction, a progeroid facial appearance, and calcification of the aortic and mitral valves. The observed valvular calcifications, located at the valve annulus, were consistent with chronic degenerative calcifications typically associated with advanced age. In our case, DXA analysis revealed a trunk-to-lower limb fat mass ratio of 0.98, with the lower limb fat mass accounting for 30.57% of the total fat mass. According to the French National Diagnosis and Care Protocol, which provides guidelines for the optimal management of Dunnigan syndrome, or familial partial lipodystrophy type 2 (FPLD2), partial lipodystrophy is defined as a trunk fat mass to lower limb fat mass ratio exceeding 1.2 or a lower limb fat mass below 25% of the total fat mass (^16^). On the basis of these criteria, our patient did not exhibit partial lipodystrophy; however, the presence of prediabetes and hypertriglyceridemia, both of which are associated with progeria, remains noteworthy. Among the progeroid syndromes linked to *LMNA* variants, Hutchinson-Gilford progeria syndrome (HGPS), atypical progeroid syndrome (APS), and mandibular dysplasia (MAD) are the most recognized phenotypes (^17^-^19^). Additionally, in 2018, Hussain and cols. described a novel phenotype associated with the *LMNA* p.T10I variant, termed generalized lipodystrophy-associated progeroid syndrome (GLPS), further expanding the spectrum of progeroid syndromes (^20^). These syndromes demonstrate variability in clinical presentation, including the age of onset, skeletal deformities, skin manifestations, degree of lipodystrophy, and severity of metabolic complications. Notably, valvulopathies related to aortic and mitral valve calcifications have been documented in HGPS and APS phenotypes (^21^). Our case differs from the MAD and HGPS phenotypes because of the absence of severe skeletal deformities, such as clavicular hypoplasia, joint contractures, and acroosteolysis, as well as the lack of skin pigmentation changes and complete alopecia. Additionally, the later onset of symptoms distinguishes our patient from those with these phenotypes. Instead, our case aligns more closely with APS, which is characterized by partial or generalized lipodystrophy and, in some cases, normal fat distribution, with a later onset of symptoms. However, APS phenotypes have not been associated with hypogonadism, setting our case apart (^21^).

The heterozygous c.331G>A p.(Glu111Lys) variant in the *LMNA* gene identified in our patient has been previously reported in the literature. Garg and cols. described this variant in a 14-year-old male within a series of 11 cases classified as atypical progeroid syndrome (^21^). Similarities between our case and the previously reported patient include tricuspid regurgitation with cardiac involvement, facial dysmorphism characterized by prominent eyes and micrognathia, and the absence of significant lipodystrophy. However, unlike the case with our patient, diabetes was not reported in the previously documented case.

In conclusion, we report a unique case of a patient who presented with secondary amenorrhea, prediabetes, and secondary osteoporosis and was found to have a *de novo* variant in exon 1 of the *LMNA* gene. This case demonstrates clinical overlap with Malouf syndrome while also exhibiting additional progeroid features, representing a distinct laminopathy. Although a previous case with this genotype-phenotype relationship has been classified as atypical progeroid syndrome, our case aligns more closely with Malouf syndrome and does not fully correspond to other *LMNA* variant-associated progeroid syndromes reported in the literature. We believe that the distinct characteristics of our case contribute valuable insights to the expanding understanding of laminopathies.

## Data Availability

the original contributions presented in the study are included in the article. Further inquiries can be directed to the corresponding author.
